# “YOU MUST BE LISTENED TO”: FACILITATORS FOR BABY BOOMERS’ SOCIAL PARTICIPATION IN CANADA AND SPAIN

**DOI:** 10.1093/geroni/igae098.1967

**Published:** 2024-12-31

**Authors:** Dolores Majón Valpuesta, Melanie Levasseur

**Affiliations:** Université de Sherbrooke, Sherbrooke, Quebec, Canada; Université de Sherbrooke, Sherbrooke, Quebec, Canada

## Abstract

In Quebec (Canada) and Spain in 2021, the baby boomers represented more than a quarter (26%) of the population. To better support a shift in healthcare and social services, this cohort requires a reassessment of their social participation, a key determinant of healthy and active aging. Despite their committement to participation, little knowledge about what influence the social participation of this generation. This presentation aims to report results of two qualitative studies exploring the facilitators for social participation in the old age of the baby boom generation. These two studies were both carried out in Spain and Quebec with 53 individual and 12 group interviews, involving a total of 107 baby boomers and 52 community representatives. Perceived facilitators were related to: a) accessibility (welcoming environment, physical and emotional proximity, virtual to overcome transportation difficulties); b) availability (flexible participation without high commitment, from a critical viewpoint towards the responsibility of cares); c) positive image (being recognized, required and respected in intergenerational settings); d) socioeconomic and health status (low cost of activities, adaptation to limitations focusing on what you can still do); and e) motivation (feeling useful, helping others and impacting on the society, deciding freely according to their values, but questioning productive ageing models). The challenges associated with supporting attractive forms and opportunities of participation adapted to the needs of this new generation were also highlighted. Responding to these challenges implies creating spaces for interactive exchanges between the baby boomers and key social agents, with real translation in health promotion policies.

